# A Social Media Campaign and Web-Based Survey About Prostate Cancer Genetics: Mixed Methods Study

**DOI:** 10.2196/69787

**Published:** 2025-10-14

**Authors:** Amy E Leader, Stacy Loeb, Preethi Selvan, Ashley Hunter, Rebecca Hartman, Scott W Keith, Veda N Giri

**Affiliations:** 1 Thomas Jefferson University Philadelphia, PA United States; 2 NYU Langone Health New York, NY United States; 3 Prostate Cancer Foundation Santa Monica, CA United States; 4 Yale Cancer Center New Haven, CT United States

**Keywords:** prostate cancer, genetics, genetic testing, social media, men’s health

## Abstract

**Background:**

Germline genetic variants are important for prostate cancer (PCa) management and hereditary cancer risk assessment, but testing is underused. Furthermore, patients are often unaware of the genetic connections to PCa. Social media is increasingly serving as a source of awareness for health information and a method to gather data from a large population.

**Objective:**

There were three objectives: to (1) create and test social media messages related to PCa genetics and genetic testing, (2) determine which social media message was most engaging, and (3) assess knowledge of and attitudes toward PCa genetic testing through an online survey using the most engaging social media message.

**Methods:**

A paid social media campaign was developed to disseminate targeted messages about PCa and genetics. We tested combinations of 8 images and 8 messages that were created or selected by the research team and reviewed by a study-specific advisory board. We targeted men and women older than 35 years living in the United States. The campaign was launched on Facebook for 6 days (June 3-8, 2023). We tracked the reach and impressions of each post. The survey, administered directly after someone viewed a post, assessed knowledge about PCa and cancer genetics as well as beliefs about cancer risk and genetic testing. Descriptive and multivariable analyses were used to analyze survey data.

**Results:**

Most posts were viewed by women (13,675/16,224, 84.3% of impressions) and people over the age of 55 years (19,997/22,906, 87.3% of impressions). The 2 most engaging images were a group of men of different races and ethnicities (reach: 28,151 people; impressions: 33,727 views), followed by a Hispanic family (reach: 16,026 people; impressions: 20,113 views). The following message had the most engagement: “Breast cancer and prostate cancer may be related because they can arise from the same gene mutation in a family” (reach: 58,980 people; impressions: 74,834 views). A total of 875 people (n=796, 91% male; mean age 43.42, SD 14.1 years; n=224, 25.6% Black or African American individuals; n=255, 29.1% Hispanic individuals) completed the survey. In total, 75.2% (658/875) strongly or somewhat agreed that genetics play a role in the development of PCa, and 84% (735/875) would want to know if they had a genetic predisposition to PCa.

**Conclusions:**

It is feasible to use social media platforms to test and disseminate messages that raise awareness about PCa genetics and the connection with other cancers (eg, breast cancer), as well as to deploy surveys that reach a wide audience.

## Introduction

### Prostate Cancer Genetics

The American Cancer Society estimated that more than 299,010 men would be diagnosed with prostate cancer (PCa) and more than 35,000 would die of PCa in 2024 [1]. Approximately 11% to 17% of patients with metastatic PCa have been reported to have germline mutations in key hereditary cancer genes, including *BRCA2*, *ATM*, *CHEK2*, *BRCA1*, *RAD51D*, or *PALB2* [2-4], which represents a substantial population burden. Patients with early-stage disease have rates of germline mutations reported at 5% to 7% [4,5]. Clinical genetic testing for PCa has rapidly expanded due to the implications for precision therapy in the metastatic setting and PCa screening [4,6-8]. Many of the genes recommended for testing confer hereditary cancer risk for individuals and their families, such as for hereditary breast and ovarian cancer or Lynch syndrome [4,6-8]. Many genes of moderate penetrance that are increasingly being tested in PCa multigene panels can provide information for targeted therapy eligibility and have additional associated cancer risks impacting patients and their families, such as *CHEK2* (breast and colorectal cancer risk), *PALB2* (breast and pancreatic cancer risk), and *ATM* (breast and pancreatic cancer risk) [4,6,7]. Therefore, the impact of genetic testing for PCa has significantly increased for patients with PCa and at risk of PCa and has expanded impact based on hereditary cancer risk for patients and their families.

### Low Understanding of PCa Genetics

Multiple factors, including feeling susceptible to PCa and understanding that PCa early detection is important, have been linked to intentions to undergo PCa genetic testing [9]. Previous research has reported low levels of knowledge among men about PCa, risk factors, screening, and genetic testing options [10,11]. Similarly, negative beliefs about the importance of cancer screening or genetic testing, as well as the efficacy of such tests, have hampered efforts to increase screening and testing for PCa among eligible men [12,13]. Disparities are most pronounced among male African American individuals, those with less formal education, and those living in rural areas [14,15].

### Social Media Can Raise Awareness of PCa Genetics

The use and role of social media in PCa continues to expand, providing a potential platform through which to promote greater knowledge and awareness [16]. With respect to PCa genetics, our group previously examined activity related to *BRCA* and genetic testing on Facebook, YouTube, and Twitter (subsequently rebranded X) [17]. Overall, we found substantially less social media activity on these topics for PCa compared to breast cancer. In a follow-up study using data from the National Cancer Institute’s Health Information National Trends Survey, we examined differences in awareness and uptake of genetic testing among participants with a history of breast or ovarian cancer versus PCa [18]. Significantly fewer patients with PCa were aware of or had used cancer genetic testing than participants with a history of breast or ovarian cancer. Interestingly, patients with PCa reported that the internet was the most common source of information about genetic testing, suggesting that this is a promising avenue to increase knowledge and awareness of this topic.

### Study Purpose

Recognizing the low awareness of PCa genetics among the public and the promise of social media as a platform for education, we developed and implemented a social media-sponsored advertising campaign with two goals: (1) to determine the images and messages about PCa genetics that resonated the most with a targeted social media audience and (2) to understand current knowledge of and attitudes toward PCa genetics among a diverse sample of respondents.

## Methods

### Study Team

This research was guided by a multidisciplinary team consisting of a medical oncologist specializing in cancer genetics, a urologist specializing in PCa, a population science researcher with training in health communication, a staff member at the Prostate Cancer Foundation, a research coordinator, a data analyst, and a biostatistician. In addition, the team was supported by a 12-member advisory board consisting of 9 clinicians who specialize in various aspects of treating PCa, a genetic counselor, a chief social media officer for a large health system, and a program director who runs a patient support group for men with PCa.

### Creating the Social Media Posts

**Figure 1:**
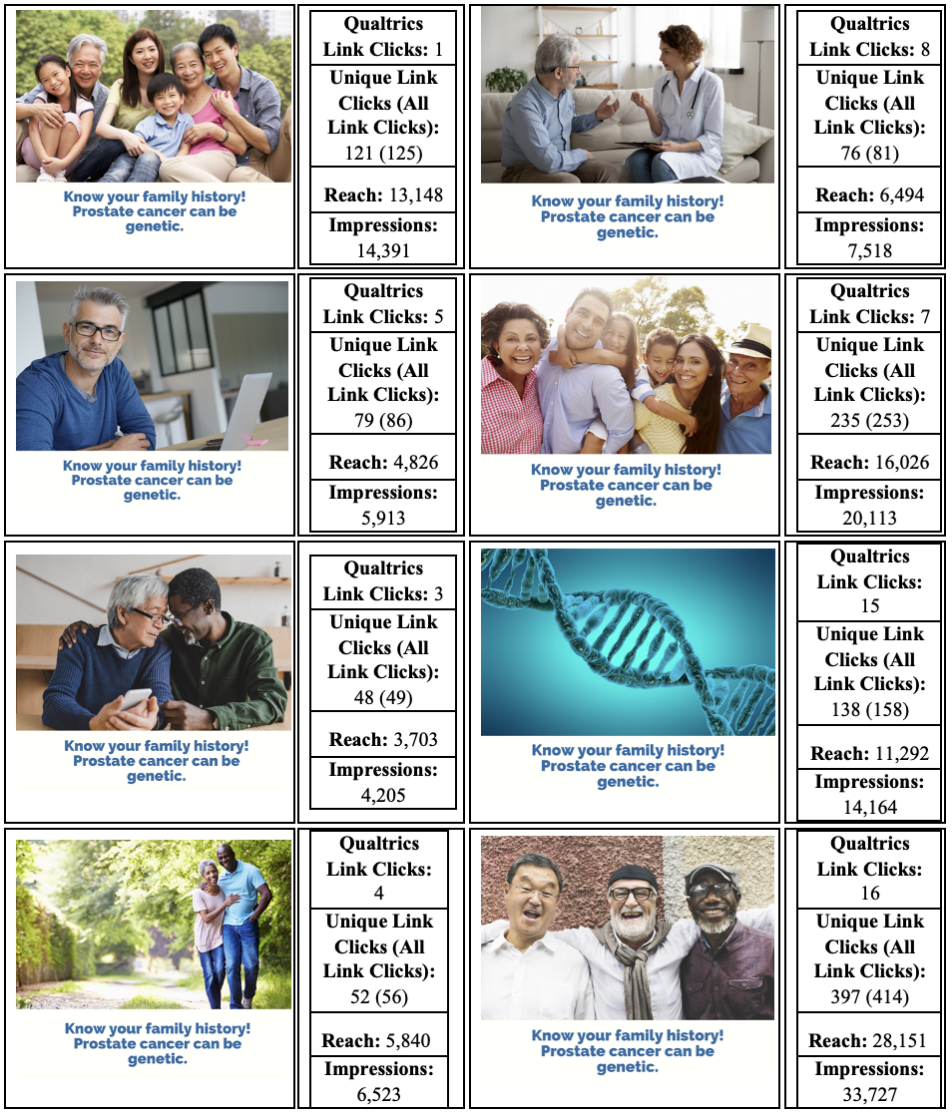
Image testing and engagement results.

**Figure 2:**
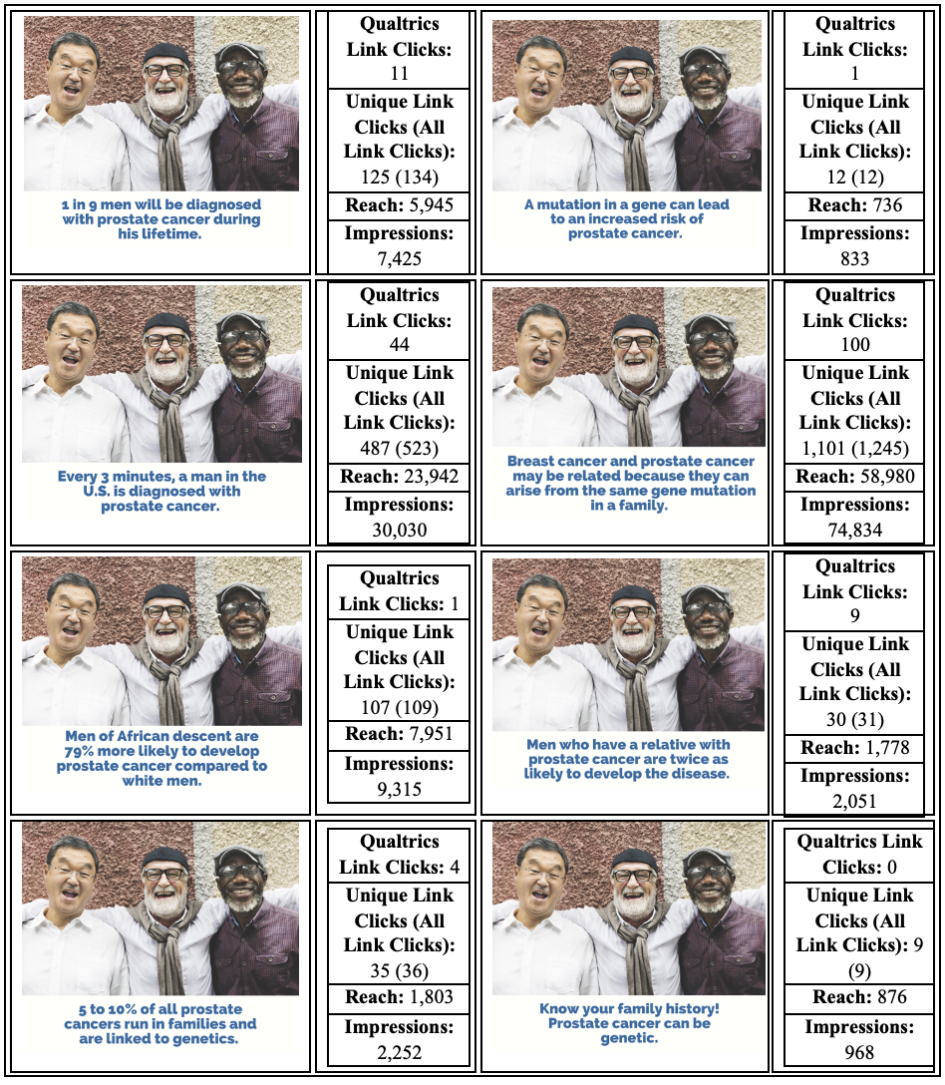
Message testing and engagement results.

The research team identified 8 different types of images that were related to PCa genetic testing: men (in general), male-female relationships, doctor-patient relationships, individuals that identify as belonging to sexual and gender minority groups, Hispanic individuals, Asian individuals, a strand of DNA, and a teal ribbon for PCa advocacy (Figure 1). Images were gathered from Shutterstock (Shutterstock, Inc). Next, the team created 8 short messages that included information about PCa statistics, its link to breast cancer, and the genetic aspect of the connection (Figure 2). All images and messages were reviewed and approved by the research team and the advisory board, which was assembled specifically for this study.

### Advertisement Placement and Targeted Audience

We used Facebook Ads Manager’s (Meta Platforms) manual advertisement placement feature to place advertisements throughout Facebook. The date and time when advertisements were shown on Facebook were determined by an algorithm that maximized viewership, although each advertisement received the same amount of placement time. We limited the viewing to male and women aged 35 years or older who resided in the United States. Although the advertisements were about PCa, we included women in our targeted audience as they often make health care decisions for spouses and family members [19].

### Metrics

To determine which image and message had the greatest engagement, we tracked metrics for each post through Facebook analytics: reach (the number of unique individuals who viewed an advertisement), impressions (the total number of people who viewed an advertisement), and total and unique number of clicks. We used the survey service platform Qualtrics (Qualtrics International Inc) to track the number of participants who clicked a link in the post that was unique to each of the 8 post combinations (Qualtrics link clicks).

### Image and Message Testing

The image and message testing phases took place over 10 days on Facebook. For the image testing, we chose the most generic message to test with each of the images (“Know your family history. Prostate cancer can be genetic”). The image with the most Qualtrics link clicks, reach, and impressions was then used to test each of the 8 messages.

### Final Campaign

**Figure 3:**
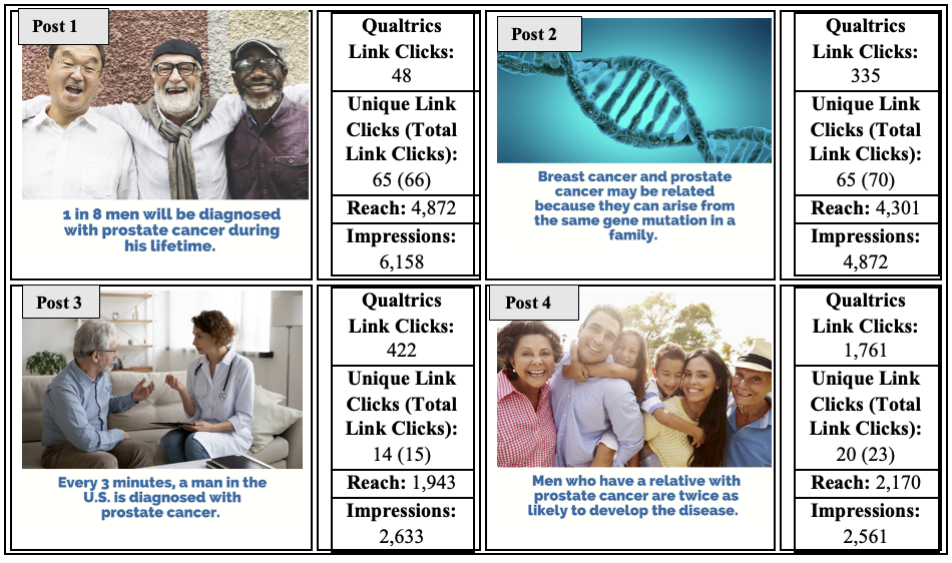
Final campaign: combination of best-performing images and messages.

The final 4 posts were created using the 4 best-performing images and messages (Figure 3). The combination of images and messages was randomly generated to create the 4 final posts. The posts depicted men of different ethnic backgrounds paired with the message “1 in 8 men will be diagnosed with prostate cancer during his lifetime” (post 1), an image of a DNA helix paired with the message “Breast cancer and prostate cancer may be related because they can arise from the same gene mutation in a family” (post 2), an image of a patient with a health care provider paired with the message “Every 3 minutes, a man in the U.S. is diagnosed with prostate cancer” (post 3), and a Hispanic family paired with the message “Men who have a relative with prostate cancer are twice as likely to develop the disease” (post 4). Each of the 4 posts included a link to the short survey about PCa genetics.

### Survey Development

We created a survey that covered the following topics: 4 questions about general knowledge of PCa and 6 questions about knowledge of PCa genetics [20], 4 questions about attitudes toward and beliefs about PCa and PCa genetic testing [21], 5 questions about family and personal history of PCa and genetic testing and demographic characteristics (sex, age, race, ethnicity, marital status, and educational attainment). The 23-item survey, with 3 additional questions if a participant indicated a PCa diagnosis, was completed, on average, in 7 (SD 1.21) minutes. Participants were required to answer each question. The survey was hosted on the Qualtrics platform. The survey is available in Multimedia Appendix 1.

### Recruitment

We launched the posts in collaboration with the Prostate Cancer Foundation and used their Facebook platform to distribute the posts over the course of 6 days (June 3-8, 2023). We paid to have the posts displayed to users whose profiles identified them as a man or woman who was over the age of 35 years and lived in the United States. These were the same criteria we used for survey eligibility. Participants clicked on a link in the social media post to verify their eligibility and take the online survey.

### Data Cleaning and Analysis

We accepted the first survey completed from each unique IP address to limit responses from spammers and bots and duplicate responses. The survey data were tallied using descriptive statistics. Mean scores with corresponding SDs were reported for the scores of correct responses for the sections on general PCa knowledge, genetics of PCa, and cancer and genetic testing beliefs. Frequencies and percentages were also reported for the number of correct responses about general PCa knowledge and genetics of PCa, and for participants’ PCa or genetic testing history and general demographic information. Multiple linear regression modeling was used to compare the mean scores between the different demographic and cancer history groups. The models were adjusted for potential confounding from age, sex, race, ethnicity, and educational level. All statistical analyses were conducted using SAS (version 9.4; SAS Institute). A nominal significance level of α=.05 was used in these analyses.

### Ethical Considerations

This study was approved by the institutional review board at Thomas Jefferson University (20G.013). Participants provided consent before completing the online survey, which consisted of reading a statement about the study and its risks and benefits and checking a box stating that they agreed to participate. All study data were anonymous and separated from the personal information required to receive US $10 compensation in the form of an e-gift card for survey completion.

## Results

### Image Testing

The 4 images with the most engagement on social media were those of men of different ethnic backgrounds, the Hispanic family, the Asian family, and the DNA helix. Each reached more than 11,000 individuals and had more than 14,000 impressions, twice as high or more than the other images. Men engaged with the posts more than women, as did those over the age of 65 years compared to younger viewers (data not shown).

### Message Testing

The following messages had the most engagement: “Breast cancer and prostate cancer may be related because they can arise from the same gene mutation in a family,” “Every 3 minutes, a man in the U.S. is diagnosed with prostate cancer,” “1 in 9 men will be diagnosed with prostate cancer during his lifetime,” and “Men who have a relative with prostate cancer are twice as likely to develop the disease.” The metrics and corresponding data can be found in Figure 2. Women were more likely to respond to the messages in the posts, as were those aged 65 years and older compared to younger viewers (data not shown).

### Final Campaign

The post with the greatest reach was the image of the men of different ethnic backgrounds accompanied by the message “1 in 8 men will be diagnosed with prostate cancer during his lifetime,” followed by an image of a DNA strand and the message “Breast cancer and prostate cancer may be related because they can arise from the same gene mutation in a family.” The doctor-patient image with the message “Every 3 minutes, a man in the U.S. is diagnosed with prostate cancer” was the third most engaging, while the fourth most engaging post was the Hispanic family with the message “Men who have a relative with prostate cancer are twice as likely to develop the disease.” Data from all 4 final posts can be found in Figure 3. The advertisements were viewed the most by women (13,675/16,224, 84.3% of impressions) and people over the age of 55 years (19,997/22,906, 87.3% of impressions; data not shown).

### Survey Participants

Of the 875 survey participants, most were male (n=796, 91%) and married or living with someone (n=701, 80.1%). The mean age of the respondents was 43.42 (SD 14.1) years; one-quarter (n=224, 25.6%) self-identified as Black or African American individuals, and almost one-third (n=255, 29.1%) self-identified as Hispanic individuals. One-third (n=302, 34.5%) had a history of PCa; of those, more than half (171/302, 56.6%) had undergone genetic testing. Of the 124 respondents who did not undergo genetic testing, 69 (55.6%) said that it was not offered to them. The characteristics of the participants are shown in Table 1.

**Table 1:** Survey respondent demographic characteristics and personal history of prostate cancer (PCa; N=875).

Variable		Values
**Sex, n (%)**		
	Male	796 (91)
	Female	79 (9)
Age (y), mean (SD)		43.42 (14.1)
**Age category (y), n (%)**		
	35-39	404 (46.2)
	40-49	160 (18.3)
	50-59	176 (20.1)
	≥60	134 (15.3)
**Race, n (%)**		
	Asian, Pacific Islander, and Alaska Native or American Indian	74 (8.5)
	Black or African American	224 (25.6)
	White	523 (59.8)
	Prefer not to answer or unknown	54 (6.2)
**Hispanic or Latino, n (%)**		
	Yes	255 (29.1)
	No	620 (70.9)
**Married or living with someone, n (%)**		
	Yes	701 (80.1)
	No	174 (19.9)
**Educational level, n (%)**		
	Lower than an associate’s degree or no college education	123 (14.1)
	Associate’s or bachelor’s degree	555 (63.4)
	Master’s or doctoral degree	197 (22.5)
**History of PCa, n (%)**		
	Yes	302 (34.5)
	No	570 (65.1)
	Do not know or unsure	3 (0.3)
**Underwent genetic testing if t** **hey had a history of** **PCa** **(n=302)** **, n (%)**		
	Yes	171 (56.6)
	No	124 (41.1)
	Missing	7 (2.3)

### Survey Findings

Respondents had varying knowledge about the prostate, PCa, and PCa genetics (Table 2). Most (699/875, 79.9%) correctly answered that the prostate was located under the bladder, but 75% (656/875) of men agreed with the statement that “more men die from prostate cancer in the U.S. than from any other cancer,” which is untrue. At least 70.1% (613/875) of respondents answered each of the PCa genetics questions correctly except for 1 question about the number of genes that can increase the risk of PCa (254/875, 29%). Three-quarters of the respondents (658/875, 75.2%) strongly or somewhat agreed that genetics play a role in the development of PCa (mean score 3.21, SD 0.76), and 84% (735/875) would want to know if they had a genetic predisposition to PCa (mean score 3.13, SD 0.80). More than half (534/875, 61%) agreed that genetic testing is risky due to privacy concerns (mean score 2.99, SD 0.97). When survey results were stratified by personal history of PCa, the results were inconclusive (Table S1 in Multimedia Appendix 2).

**Table 2:** Knowledge and beliefs about prostate cancer and prostate cancer genetics (N=875).

Statement			Values
**Knowledge about** **prostate cancer**			
	“Both men and women have a prostate gland,” n (%)	411 (47)	
	“The prostate gland is located under the bladder,” n (%)	699 (79.9)	
	“Black men are more likely to get prostate cancer than White men,” n (%)	584 (66.7)	
	“More men die from prostate cancer in the U.S. than from any other cancer,” n (%)	219 (25)	
	Score (number correct), mean (SD)	2.19 (0.87)	
**Knowledge about prostate cancer genetics**			
	“People get half of their genetic makeup from their mother and half from their father,” n (%)	646 (73.8)	
	“There is only one gene that can increase the risk of prostate cancer,” n (%)	254 (29)	
	“A mutation in a gene can lead to an increased risk of cancer,” n (%)	667 (76.2)	
	“If a woman has a breast cancer gene mutation (such as BRCA2), she can pass that mutation to her son,” n (%)	649 (74.2)	
	“The breast cancer gene BRCA2 can increase the risk for prostate cancer,” n (%)	625 (71.4)	
	“Breast cancer and prostate cancer may be related because they can arise from the same gene mutation in a family,” n (%)	655 (74.9)	
	Score (number correct), mean (SD)	4.00 (1.38)	
**Beliefs about prostate cancer (score of 1-4), mean (SD)**			
	“How much do you think genetics, that is characteristics passed from one generation to the next, determine whether or not a person will develop prostate cancer?”^a^	3.21 (0.8)	
	“I would want to know if I have a genetic risk for prostate cancer”^b^	3.13 (0.8)	
	“Getting genetic testing is risky because you cannot guarantee the privacy of the results”^b^	2.99 (1.0)	

^a^Response options: 4=“a lot,” 3=“somewhat,” 2=“a little,” and 1=“not at all.”

^b^Response options: 4=“strongly agree,” 3=“agree,” 2=“disagree,” and 1=“strongly disagree.”

Multiple linear regression modeling suggested that there were notable differences in PCa and PCa genetics knowledge and beliefs that depended on the demographic characteristics of the respondents (Table 3). Lower mean scores on knowledge of PCa were associated with those reporting the oldest ages compared to those under the age of 40 years (for those aged ≥60 years: β=−.20; *P*=.024), although higher scores were associated with reporting Asian, Pacific Islander, or Alaska Native race compared to White race (β=.22; *P*=.046) or Hispanic or Latino ethnicity (β=.23; *P*=.001). Higher mean scores on knowledge of PCa genetics were associated with those reporting older ages compared to those under the age of 40 years (for those aged 40-49 years=: β.41 and *P*=.001; for those aged 50-59 years: β=.539 and *P*<.001; for those aged ≥60 years: β=.65 and *P*<.001), Black or African American race compared to White race (β=.36; *P*=.001), or higher educational attainment compared to those without a college degree (for those with an associate’s or bachelor’s degree: β=.47 and *P*<.001; for those with a master’s or doctoral degree: β=.69 and *P*<.001).

Regression modeling for additional survey items, as well as modeling for participants with and without a personal history of PCa, is shown in Tables S2-S6 in Multimedia Appendix 2. Stronger agreement with the statement “Getting genetic testing is risky because you cannot guarantee the privacy of the results” was associated with those reporting older ages compared to those under the age of 40 years (for those aged 50-59 years: β=.19 and *P*=.03; for those aged ≥60 years: β=.37 and *P*<.001), Hispanic or Latino ethnicity (β=.20; *P*=.007), or higher educational attainment (for those with an associate’s or bachelor’s degree: β=.25 and *P*=.01; for those with a master’s or doctoral degree: β=.54 and *P*<.001), although agreement was weaker for female than for men (β=−.38; *P*=.001). Stronger agreement with “How much do you think genetics, that is characteristics passed from one generation to the next, determine whether or not a person will develop prostate cancer?” was associated with those reporting older ages compared to those under the age of 40 years (for those aged 40-49 years: β=.14 and *P*=.04; for those aged 50-59 years: β=.38 and *P*<.001; for those aged ≥60 years: β=.58 and *P*<.001) or the highest educational attainment compared to lower than an associate’s degree (for those with a master’s or doctoral degree: β=.34; *P*<.001), although agreement was weaker for those reporting Hispanic or Latino ethnicity (β=−.13; *P*=.03). No direct associations in modeling by personal history of PCa were observed.

**Table 3:** Multiple linear regression model results for prostate cancer and prostate cancer genetics knowledge.

Parameter	Prostate cancer knowledge^a^				Prostate cancer genetics knowledge^b^		
	Estimate (SE)	*t* test (*df*)	*P* value	Estimate (SE)		*t* test (*df*)	*P* value
Intercept	1.955 (.083)	23.510 (1)	<001	3.202 (.126)		25.390 (1)	.001
Sex: female	.142 (.102)	1.390 (1)	.17	0.099 (0.155)		0.640 (1)	0.52
Sex: male	0	―^c^	―	0		―	―
Age: 40-49 y	0.057 (0.081)	0.690 (1)	.49	0.411 (0.124)		3.320 (1)	.001
Age: 50-59 y	−0.032 (0.080)	−0.400 (1)	.69	0.539 (0.122)		4.440 (1)	<.001
Age: ≥60 y	−0.203 (0.090)	−2.260 (1)	.02	0.652 (0.137)		4.770 (1)	<.001
Age: ≤39 y	0	―	―	0		―	―
Race: Asian, Pacific Islander, and Alaska Native	0.215 (0.107)	2.000 (1)	.05	−0.256 (0.163)		−1.570 (1)	.12
Race: Black or African American	0.135 (0.070)	1.930 (1)	.05	0.362 (0.106)		3.410 (1)	.001
Race: prefer not to answer or unknown	0.040 (0.129)	0.310 (1)	.76	−0.816 (0.195)		−4.190 (1)	<.001
Race: White	0	―	―	0		―	―
Ethnicity: Hispanic or Latino**―**yes	0.234 (0.069)	3.400 (1)	.001	0.103 (0.104)		0.980 (1)	.33
Ethnicity: Hispanic or Latino**―**no	0	―	―	0		―	―
Educational level: associate’s or bachelor’s degree	0.143 (0.088)	1.630 (1)	.10	0.469 (0.133)		3.530 (1)	<.001
Educational level: master’s or doctoral degree	0.149 (0.104)	1.430 (1)	.15	0.687 (0.157)		4.370 (1)	<.001
Educational level: lower than an associate’s or college degree	0	―	―	0		―	―

^a^Summary score of 4 items.

^b^Summary score of 6 items.

^c^Not applicable.

## Discussion

### Principal Findings

This study combined a social media advertising campaign about PCa genetics to determine the images and message text that resonated the most with an at-risk audience with a brief survey to understand knowledge and beliefs about PCa and PCa genetics. Through a series of image and message tests for engagement on social media, we created 4 posts for Facebook and linked those posts to a brief survey. Three of the 4 high-engagement images had families in them, and 3 of the 4 messages had the word “male” or “man” in them. A total of 875 people who saw a post completed the survey, leading to results that showed that knowledge about PCa and PCa genetics, as well as beliefs about PCa genetics, vary significantly by the characteristics and experiences of the individual. Results from the social media portion of this study can be used to inform future PCa genetics awareness campaigns; in fact, a recent roundtable of experts endorsed social media as an effective channel to reach patients with credible messages [22].

### Comparison With Prior Work

The image testing revealed that a variety of images—from groups of men to patients and providers to families to images of DNA—resonated with the audience. Interestingly, while the image of the Asian family had high engagement, it did not result in many completed surveys. This may be due to the reluctance of people who are of Asian descent to participate in research [23]. For all advertisements, the most engagement and interactions came from participants aged 65 years and older, consistent with data that show that 6 out 10 men are aged 65 years or older when they are diagnosed with PCa, and therefore, this age group is most likely to be looking for this information [24].

While 3 of the 4 messages with the highest engagement included the word “men” or “man,” the message with the highest engagement linked both breast cancer and PCa. It may be that this information about the connection between breast cancer and PCa is new and piqued people’s curiosity. In an environment such as social media where the volume of information and messages is infinite, new information may stand out from the noise. Notably, in the survey data, more than 7.1% (613/875) of the respondents answered 2 questions correctly about the genetic link between breast cancer and PCa, possibly correctly recalling what they had just viewed in the post. Women were more likely to engage with messages than men. The most engagement from women was with more broad, general messages. Similar to the image testing, all messages had more engagement among those aged 65 years or older. Our experience and results indicate that, when creating public information campaigns, it is important to pretest images and messages to determine which have the most salience for different audiences [25].

From the survey data, there was strong understanding that genetics can play a role in PCa development, and there was high interest in knowing one’s own risk of PCa, but there was also concern that genetic testing carries some risk with respect to data privacy. There is a substantial evidence base about the public’s understanding of PCa and the disparities in knowledge among subpopulations, but less is known about the public’s understanding of the role of genetics in the disease. While data protection and privacy is a dynamic field, there is a sense that patients are concerned about sharing their genetic data [26]. Increasing uptake of genetic testing across all populations will require continued educational campaigns coupled with information to alleviate concerns about data privacy.

### Limitations

There were numerous strengths and limitations to this study. Strengths of this study included the novel use of social media posts for an awareness campaign about PCa genetics and to conduct a survey on educational gaps in this area. Limitations of this study included that the results of a survey conducted through social media may not be generalizable to people who do not use social media platforms. It is also possible that bots may have affected the results, although checks were in place to reduce this risk. In addition, a single social media platform was used to post the advertisements, and results may differ on other platforms with different audiences.

### Conclusions

Our results show that it is feasible to use social media platforms to create effective public awareness campaigns and increase awareness of the link between PCa and genetics. Given the lack of awareness of this link and the implications for medical surveillance and the early detection of cancer, campaigns such as this one are warranted. To be effective and attention getting, messages should be tailored to different viewers, as indicated by our results. Further studies are needed to assess calls to action based on social media campaigns to increase engagement for PCa genetic testing across populations.

## Appendix

Multimedia Appendix 1 Study survey.

Multimedia Appendix 2 Supplementary tables.

## Abbreviations

PCa: prostate cancer

## Data Availability

The datasets generated or analyzed during this study are available from the corresponding author on request.
