# Supplementary material for: A Social Media Campaign and Web-Based Survey About Prostate Cancer Genetics: Mixed Methods Study
Source: JMIR Cancer. 2025 Oct 14;11:e69787. doi: 10.2196/69787 (PMC12569485; doi:10.2196/69787)
Supplement: Multimedia Appendix 1 [file cancer_v11i1e69787_app1.pdf]

1. Please answer the following questions about prostate cancer to the best of your knowledge:

Question	Response Options:
Both men and women have a prostate gland.	T/F/Don't Know
The prostate gland is located under the bladder.	T/F/Don't Know
Black men are more likely to get prostate cancer than white men.	T/F/Don't Know
More men die from prostate cancer in the U.S. than from any other cancer.	T/F/Don't Know

2. The next questions are about the genetics of prostate cancer. Please answer to the best of your knowledge.

Question	Response Options:
People get half of their genetic makeup from their mother and half from their father.	T/F/Don't Know
There is only one gene that can increase the risk of prostate cancer.	T/F/Don't Know
A mutation in a gene can lead to an increased risk of cancer.	T/F/Don't Know
If a woman has a breast cancer gene mutation (such as BRCA2), she can pass that mutation to her son.	T/F/Don't Know
The breast cancer gene BRCA2 can increase the risk for prostate cancer.	T/F/Don't Know
Breast cancer and prostate cancer may be related because they can arise from the same gene mutation in a family.	T/F/Don't Know

3. The next questions are about your and your family's health history. Please answer to the best of your knowledge.

a. Have you ever been told by a doctor that you have prostate cancer?
Yes
No
Unsure/Don't Know

If yes to Question 3a:

What year were you told that you had prostate cancer?

Yes
No
Unsure/Don't Know

b. Are you currently being treated for your prostate cancer?

Yes
No
Unsure/Don't Know

c. Did you have genetic testing for your prostate cancer?

Yes
No
Unsure/Don't Know

If yes, how was the test performed:

- Through my doctor
- I did it myself through an online/at-home genetic testing company, like 23andme.com
- Other: _______________________

If no, what is/was the primary reason for not having genetic testing?

- My doctor didn't offer it to me
- I didn't want to have genetic testing
- Other: ________________________

d. Have any of your close relatives been diagnosed with prostate cancer? Select all that apply.

Father
Grandfather
Brother
Sons
Uncles
Cousins

4. The next set of questions are about your beliefs about prostate cancer and genetic testing:

a. How much do you think genetics, that is characteristics passed from one generation to the next, determine whether or not a person will develop prostate cancer?

A lot	Somewhat	A little	Not at All

b. I would want to know if I have a genetic risk for prostate cancer.

Strongly Agree	Agree	Disagree	Strongly Disagree

c. Getting genetic testing is risky because you cannot guarantee the privacy of the results.

Strongly Agree	Agree	Disagree	Strongly Disagree

d. Compared to the average person your age, would you say that you are more likely to get prostate cancer, less likely, or about as likely? (Skip for those who answered “yes” to Q 3a).

More Likely	About as Likely	Less Likely

5. The last set of questions are about you.

a. Are you:

Male
Female
Prefer Not to Answer

b. How old are you? _____________

c. What is your race? Choose all that apply.

American Indian/Alaskan Native
Asian
Black/African American
Native Hawaiian/Pacific Islander
White
Other: ______________________

d. Are you Hispanic or Latino?

Yes
No
Prefer Not to Answer

e. Are you married or living with someone?

Yes
No

f. What is your highest level of education?

High School/Some College
Associate or Bachelor’s Degree
Master’s or Doctoral Degree
