# Supplementary material for: A Social Media Campaign and Web-Based Survey About Prostate Cancer Genetics: Mixed Methods Study
Source: JMIR Cancer. 2025 Oct 14;11:e69787. doi: 10.2196/69787 (PMC12569485; doi:10.2196/69787)
Supplement: Multimedia Appendix 2 [file cancer_v11i1e69787_app2.pdf]

Supplemental Table 1: Knowledge and beliefs about prostate cancer and prostate cancer genetics, by personal history of prostate cancer

	No PCA History		PCA History	
**Knowledge about Prostate Cancer (N=570)**	**Correct**		**Correct**	
**Question**	**N**	**%**	**N**	**%**
Both men and women have a prostate gland.	228	40.00	182	60.26
The prostate gland is located under the bladder.	465	81.58	232	76.82
Black men are more likely to get prostate cancer than white men.	403	70.70	180	59.60
More men die from prostate cancer in the U.S. than from any other cancer.	135	23.68	82	27.15
**Score** (Number Correct)	2.16 (Mean)	0.87 (SD)	2.24 (Mean)	0.85 (SD)
**Knowledge about Prostate Cancer Genetics (N=570)**				
**Question**	**N**	**%**	**N**	**%**
People get half of their genetic makeup from their mother and half from their father.	457	80.18	187	61.92
There is only one gene that can increase the risk of prostate cancer.	160	28.07	92	30.46
A mutation in a gene can lead to an increased risk of cancer.	449	78.88	215	71.19
If a woman has a breast cancer gene mutation (such as BRCA2), she can pass that mutation to her son.	439	77.02	210	69.54
The breast cancer gene BRCA2 can increase the risk for prostate cancer.	423	74.21	200	66.23
Breast cancer and prostate cancer may be related because they can arise from the same gene mutation in a family.	436	76.49	218	72.19
**Score, Number Correct** (Mean, SD)	4.15	1.34	3.72	1.41
**Beliefs about Prostate Cancer (Mean, SD)**				
How much do you think genetics, that is characteristics passed from one generation to the next, determine whether or not a person will develop prostate cancer?[1]	3.24	0.75	3.14	0.78
I would want to know if I have a genetic risk for prostate cancer.[2]	3.11	0.86	3.17	0.66
Getting genetic testing is risky because you cannot guarantee the privacy of the results.[2]	2.88	1.02	3.19	0.83

[1]Response options: *4 = A lot, 3 = Somewhat, 2 = A little, 1 = Not at all*; [2]: *4 = Strongly agree, 3 = Agree, 2 = Disagree, 1 = Strongly disagree*

Supplemental Table 2: Multiple linear regression model, belief about the role of genetics in prostate cancer development*

Parameter	Estimate	Standard Error	t Value	Pr > |t|
**Intercept**	2.851	0.074	38.610	<.001
Sex: Female	0.087	0.087	1.000	0.318
Sex: Male	0	.	.	.
Age: 40-49	0.143	0.070	2.040	0.042
Age: 50-59	0.382	0.068	5.640	<.001
Age: ≥60	0.575	0.076	7.590	<.001
Age: ≤39	0	.	.	.
Race: Asian, Pacific Islander, and Alaskan Native	0.096	0.092	1.040	0.297
Race: Black or African American	0.069	0.059	1.170	0.241
Race: Prefer not to answer/unknown	0.103	0.113	0.910	0.362
Race: White	0	.	.	.
Ethnicity: Hispanic or Latino Yes	-0.129	0.060	-2.160	0.031
Ethnicity: Hispanic or Latino No	0	.	.	.
Education: Associate's or Bachelor's Degree	0.112	0.077	1.450	0.148
Education: Masters or Doctoral degree	0.340	0.090	3.770	<.001
Education: Less than an Associate's Degree/College education	0	.	.	.

* "How much do you think genetics, that is characteristics passed from one generation to the next, determine whether or not a person will develop prostate cancer?" (4-point scale, 4 = a lot to 1 = not at all)

Supplemental Table 3: Multiple linear regression model results, interest in knowing one's genetic risk for prostate cancer*

Parameter	Estimate	Standard Error	t Value	Pr > |t|
Intercept	2.687	0.073	36.620	<.001
Sex: Female	0.061	0.090	0.670	0.501
Sex: Male	0	.	.	.
Age: 40-49	0.380	0.072	5.290	<.001
Age: 50-59	0.335	0.071	4.740	<.001
Age: ≥ 60	0.485	0.079	6.110	<.001
Age: ≤39	0	.	.	.
Race: Asian, Pacific Islander, and Alaskan Native	0.028	0.095	0.290	0.771
Race: Black or African American	-0.005	0.062	-0.090	0.929
Race: Prefer not to answer/unknown	0.147	0.113	1.290	0.197
Race: White	0	.	.	.
Ethnicity: Hispanic or Latino Yes	-0.194	0.061	-3.180	0.002
Ethnicity: Hispanic or Latino No	0	.	.	.
Education: Associate's or Bachelor's Degree	0.290	0.077	3.750	<.001
Education: Masters or Doctoral degree	0.408	0.092	4.460	<.001
Education: Less than an Associate's Degree/College education	0	.	.	.

* "I would want to know if I have a genetic risk for prostate cancer." (4-point scale, 4 = strongly agree to 1 = strongly disagree)

Supplemental Table 4: Multiple linear regression model results, belief that genetic testing is risky*

Parameter	Estimate	Standard Error	t Value	Pr > |t|
Intercept	2.585	0.091	28.540	<.001
Sex: Female	-0.375	0.111	-3.380	0.001
Sex: Male	0	.	.	.
Age: 40-49	0.165	0.089	1.860	0.063
Age: 50-59	0.193	0.087	2.210	0.028
Age: ≥ 60	0.373	0.098	3.810	<.001
Age: ≤39	0	.	.	.
Race: Asian, Pacific Islander, and Alaskan Native	-0.015	0.117	-0.130	0.900
Race: Black or African American	-0.106	0.076	-1.390	0.164
Race: Prefer not to answer/unknown	0.022	0.140	0.160	0.874
Race: White	0	.	.	.
Ethnicity: Hispanic or Latino Yes	0.204	0.075	2.710	0.007
Ethnicity: Hispanic or Latino No	0	.	.	.
Education: Associate's or Bachelor's Degree	0.245	0.095	2.560	0.011
Education: Masters or Doctoral degree	0.542	0.113	4.790	<.001
Education: Less than an Associate's Degree/College education	0	.	.	.

* "Getting genetic testing is risky because you cannot guarantee the privacy of the results." (4 point scale, 4 = strongly agree to 1 = strongly disagree)

Supplemental Table 5. Multiple linear regression model results, prostate cancer knowledge and prostate cancer genetics knowledge, by no personal history of prostate cancer

	Prostate Cancer Knowledge				Prostate Cancer Genetics Knowledge			
**Parameter**	**Estimate**	**Standard Error**	**t Value**	**Pr > |t|**	**Estimate**	**Standard Error**	**t Value**	**Pr > |t|**
Intercept	1.900	0.090	21.15	<.001	3.145	0.131	23.93	<.001
Sex: Female	0.059	0.112	0.53	0.597	-0.077	0.164	-0.47	0.64
Sex: Male	0	.	.	.	0	.	.	.
Age: 40-49	-0.039	0.105	-0.37	0.712	0.256	0.154	1.66	0.10
Age: 50-59	-0.085	0.103	-0.82	0.410	0.354	0.150	2.35	0.02
Age: ≥ 60	-0.278	0.109	-2.54	0.011	0.494	0.160	3.09	<.001
Age: ≤39	0	.	.	.	0	.	.	.
Race: Asian, Pacific Islander, and Alaskan Native	0.145	0.148	0.98	0.330	-0.487	0.217	-2.25	0.02
Race: Black or African American	0.173	0.086	2.02	0.043	0.255	0.125	2.03	0.04
Race: Prefer not to answer/unknown	-0.170	0.208	-0.81	0.416	-0.574	0.305	-1.88	0.06
Race: White	0	.	.	.	0	.	.	.
Ethnicity: Hispanic or Latino: Yes	0.386	0.097	3.99	<.001	0.513	0.142	3.63	<.001
Ethnicity: Hispanic or Latino: No	0	.	.	.	0	.	.	.
Education: Associate or bachelor's degree	0.219	0.099	2.22	0.027	0.805	0.144	5.58	<.001
Education: Masters or doctoral degree	0.280	0.118	2.38	0.018	0.996	0.172	5.78	<.001
Education: Less than an associate's/college degree	0	.	.	.	0	.	.	.

Supplemental Table 6. Multiple linear regression model results, prostate cancer knowledge and prostate cancer genetics knowledge, by having a personal history of prostate cancer

History of Prostate Cancer

	Prostate Cancer Knowledge				Prostate Cancer Genetics Knowledge			
**Parameter**	**Estimate**	**Standard Error**	**t Value**	**Pr > |t|**	**Estimate**	**Standard Error**	**t Value**	**Pr > |t|**
Intercept	2.217	0.248	8.94	<.001	3.145	0.375	8.38	<.001
Sex: Female	0.470	0.275	1.71	0.088	-0.416	0.416	-1	0.319
Sex: Male	0	.	.	.	0	.	.	.
Age: 40-49	0.163	0.130	1.25	0.212	0.698	0.197	3.54	0.001
Age: 50-59	0.039	0.129	0.3	0.763	0.975	0.195	5	<.001
Age: ≥ 60	-0.081	0.159	-0.51	0.610	0.898	0.241	3.73	<.001
Age: ≤39	0	.	.	.	0	.	.	.
Race: Asian, Pacific Islander, and Alaskan Native	0.188	0.159	1.18	0.237	0.094	0.240	0.39	0.695
Race: Black or African American	0.090	0.123	0.73	0.465	0.622	0.186	3.34	0.001
Race: Prefer not to answer/unknown	0.149	0.165	0.9	0.366	-0.762	0.250	-3.05	0.003
Race: White	0	.	.	.	0	.	.	.
Ethnicity: Hispanic or Latino: Yes	0.095	0.104	0.91	0.362	-0.177	0.157	-1.13	0.261
Ethnicity: Hispanic or Latino: No	0	.	.	.	0	.	.	.
Education: Associate or bachelor's degree	-0.113	0.238	-0.48	0.635	0.082	0.360	0.23	0.821
Education: Masters or doctoral degree	-0.230	0.261	-0.88	0.380	0.163	0.396	0.41	0.681
Education: Less than an associate's/college degree	0	.	.	.	0	.	.	.
